# Supplementary figures and images for: Humans differ in their personal microbial cloud
Source: PeerJ. 2015 Sep 22;3:e1258. doi: 10.7717/peerj.1258 (PMC4582947; doi:10.7717/peerj.1258)

➤ Air Flow Direction  
— Visqueen  
▨ Plenum

◀ Air Filter  
● Settling Dish  
◆ Particle Counter

(a)

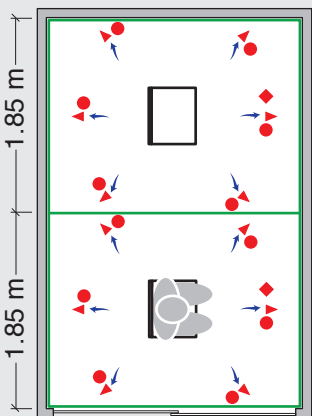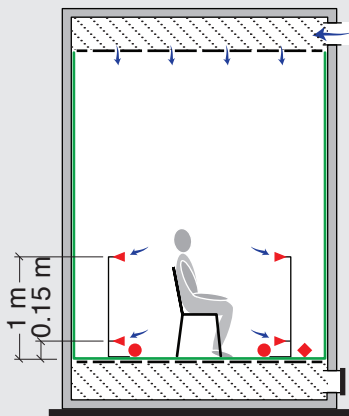

(b)

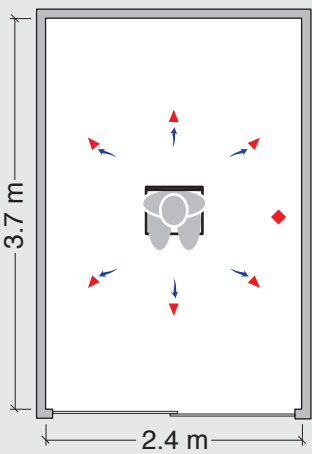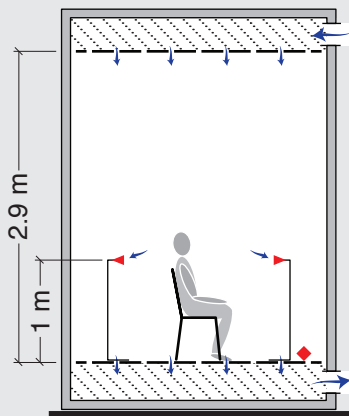

Supplement: Figure S1 — Schematic of the experimental chamber during both experiments. (a) The objective of the first experiment was to distinguish occupied from unoccupied airborne bacterial communities. Thus the test chamber was split into two identical portions, and air was collected on both sides simultaneously. Supply air velocity (entering through the ceiling plenum) was determined to replace the volume of air removed by vacuum sampling, as well as create slight positive pressure within the occupied chamber. (b) The second experiment was designed to distinguish among occupants, so the test chamber was not divided, but rather vacuum samples were taken in the supply ventilation system, surrounding the occupant in the chamber, and also in the exhaust ventilation system. During the second experiment, supply air velocity resulted in 1 & 3 ACH. [file peerj-03-1258-s001.pdf]

## (a) Air Filters

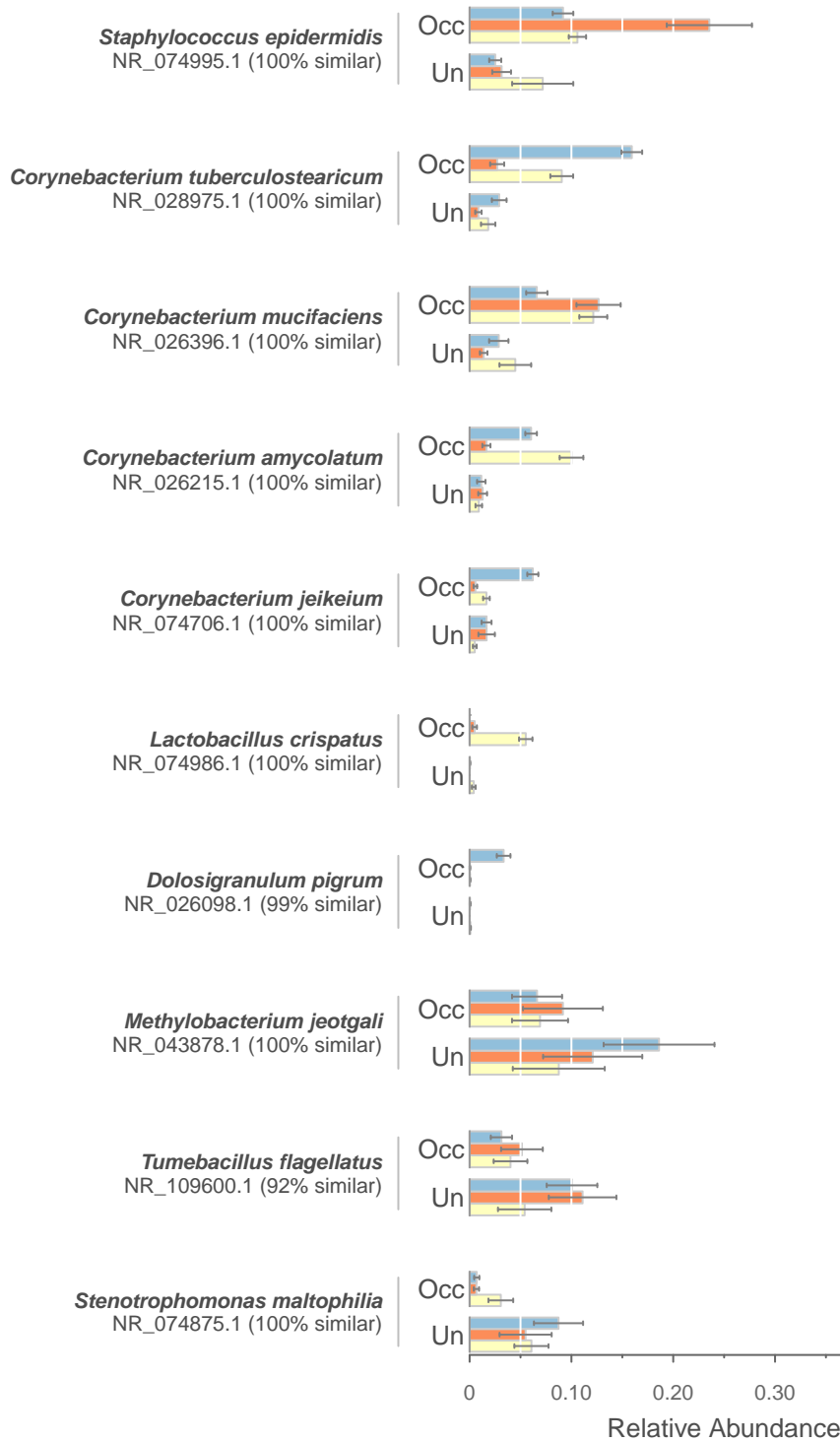

## (b) Settling Dishes

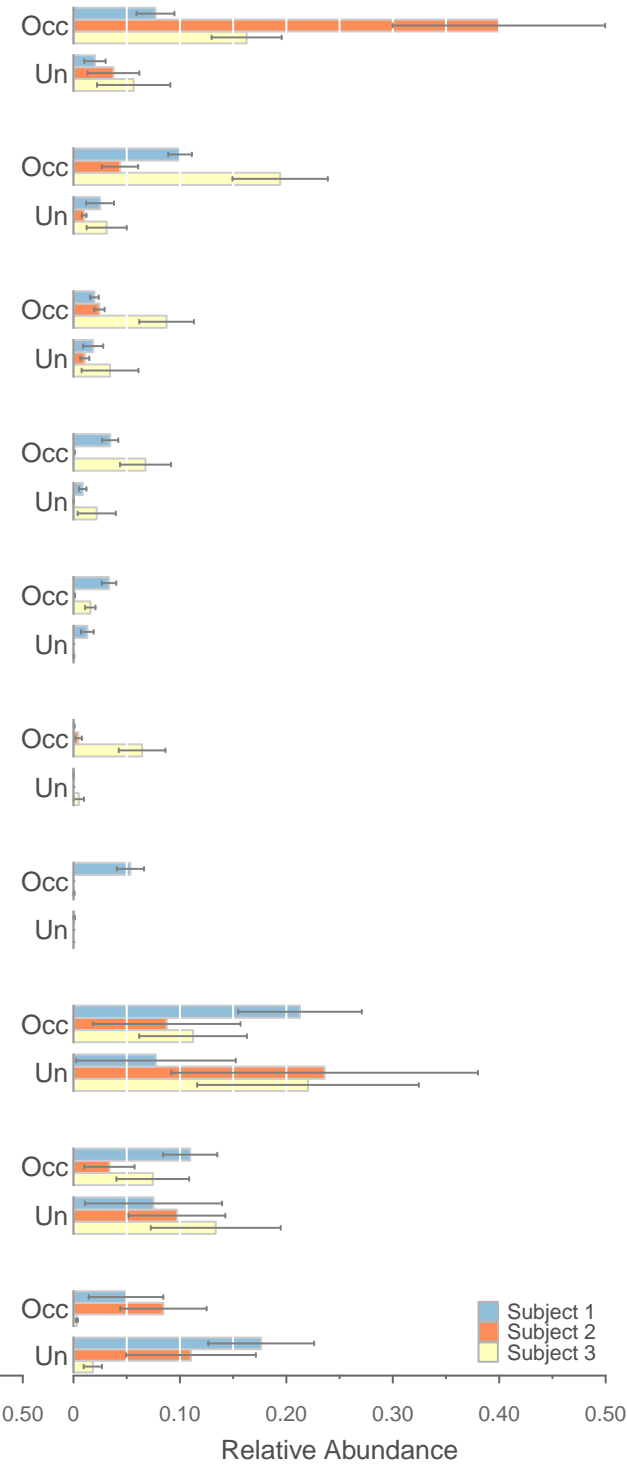

Supplement: Figure S2 — Indicator OTUs from the first experiment were consistent for occupants regardless of sampling method. (a) The most abundant OTUs in 4-hour air filters were clearly indicative of either occupied air (top seven OTUs) or unoccupied air (bottom three OTUs), regardless of occupant. The top OTU (Staphylococcus epidermidis) was the most abundant OTU found in occupied samples. (b) These same OTUs were also consistently abundant in settling dishes. Bars are mean relative abundance, and error bars show ±1 standard error; the number of replicates in each treatment is detailed in Table 2. Eight of these ten OTUs were also significant indicator taxa included in Table 3. [file peerj-03-1258-s003.pdf]

## Occupied

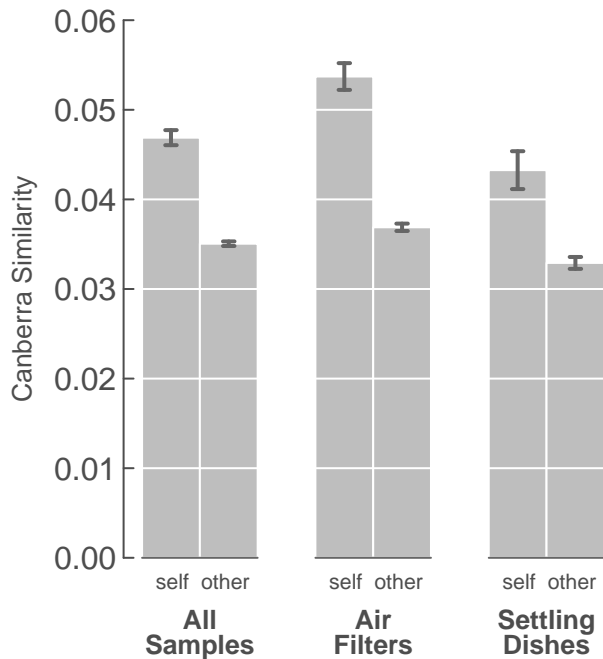

## Unoccupied

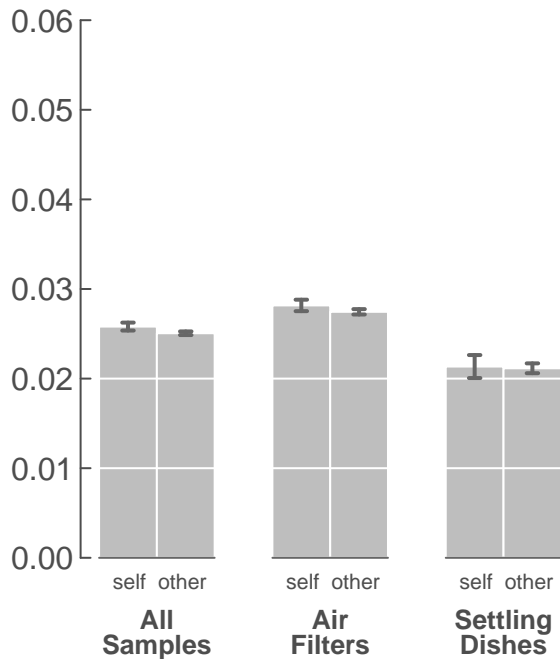

Supplement: Figure S3 — Significant differences among occupant personal microbial clouds are not explained by temporal changes in background airborne microbial assemblages. We detected marginal differences in background bacteria (i.e., day-to-day temporal changes). These differences, however, were negligible when compared to differences among the different occupants. (Left) Community similarities in the left plot (occupied samples from the first experiment; same data as shown in Fig. 1E) show that occupants were more similar to other samples from the same person than to other occupants, regardless of sampling method. This difference was significantly more pronounced than that of unoccupied samples (right) taken simultaneously during sampling periods. Error bars represent ±1 standard error on pairwise Canberra similarities. [file peerj-03-1258-s004.pdf]

1 Air Change / Hour

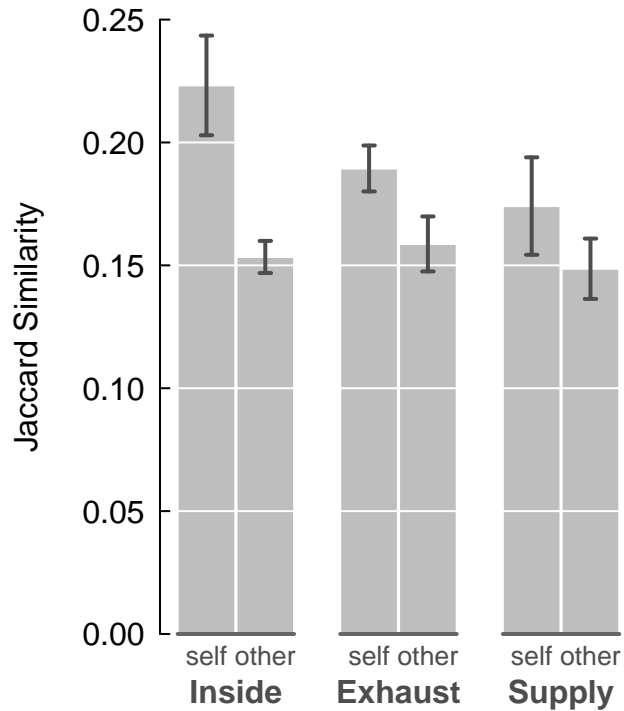

3 Air Changes / Hour

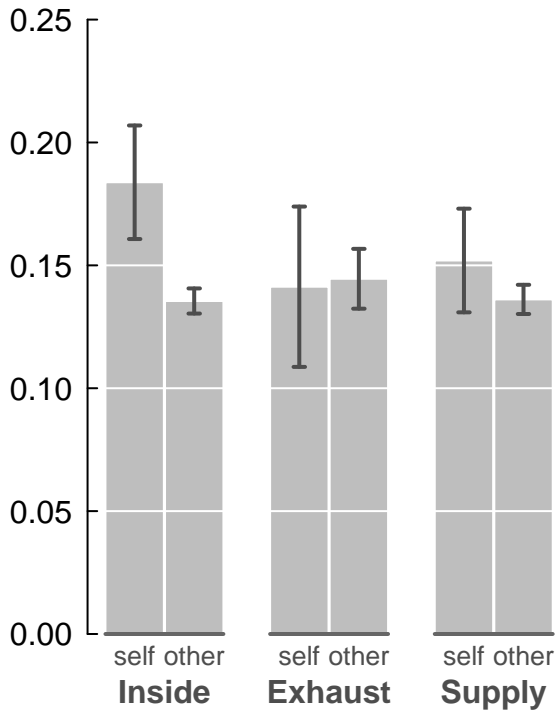

Supplement: Figure S4 — At 1 air change per hour (left), occupants were, on average, detectable inside the chamber, but less so in exhaust air. When air exchange rates were tripled (right), these signals disappeared, and not a single occupant was consistently detectable, even in occupied indoor air. Bars show average Jaccard Similarity values ±1 standard error. [file peerj-03-1258-s005.pdf]
