# Supplementary material for: Humans differ in their personal microbial cloud
Source: PeerJ. 2015 Sep 22;3:e1258. doi: 10.7717/peerj.1258 (PMC4582947; doi:10.7717/peerj.1258)
Supplement: Table S1 [file peerj-03-1258-s002.pdf]

Supporting Tables

Table S1: Particle counts during the first experiment generally increased with occupation over 4 hours.

	10+ μm*	5-10 μm	2.5-5 μm
Subject 1	14.74	2.58	1.66
Subject 2	29.29	4.31	2.31
Subject 3	1.84	0.99	0.96
mean	15.29	2.63	1.64

* All counts are expressed as ratios of particles L^{-1} minute^{-1} above unoccupied controls.
